# Supplementary material for: Diagnostic Values of Red Flags and a Clinical Prediction Score for Emergent Intracranial Lesions in Non-Traumatic Pediatric Headaches
Source: Children (Basel). 2022 Jun 10;9(6):863. doi: 10.3390/children9060863 (PMC9221978; doi:10.3390/children9060863)
Supplement: Supplementary file 1 [file children-09-00863-s001.zip › children-1723067-supplementary.pdf]

Supporting information Table S1 Emergent and non-emergent intracranial lesions:

Emergent	Non-emergent
Brain tumors	White matter abnormalities associated with migraines
Meningitis (i.e. viral or bacterial)	Pineal cyst
Encephalitis (i.e. infective or autoimmune associated)	Calcified granuloma
Indiopathic intracranial hypertension	Choroid plexus xanthogranuloma
Intracranial hemorrhage (i.e. SAH, SDH, EDH or intraparenchymal hemorrahge)	Developmental venous anomaly
Reversible cerebral vasoconstriction syndrome (RCVS)	Mild sinusitis
Posterior reversible encephalopathy syndrome (PRES)	Cavum septi pellucidi, cavum vergae or cavum velum interpositum
Cerebral venous sinus thrombosis	
Shunt malfunction	
Ischemic stroke	
Hydrocephalus	
Multiple sclerosis (MS)	
Major congenital malformation/ anomaly (i.e. Dandy-Walker malformation, Chiari malformation, Sturge-Weber syndrome & PHACES)	
Cystic brain lesion with mass effect or hydrocephalus (i.e. colloid cyst with hydrocephalus or arachnoid cyst with mass effect to cerebral hemisphere)	
Cavernous malformation (particularly bleeding lesion)	
Invasive fungal sinusitis with intracranial extension	

Supporting information Table S2 The Final Diagnosis of the Pediatric Patients with Non-Traumatic Headaches (N=109)

Diagnosis	Frequency	Percentage
Emergent lesion		
Brain tumor	13	11.93%
Intracranial infection	13	11.93%
Intracranial hemorrhage	7	6.42%
Reversible cerebral vasoconstriction syndrome (RVCS)	3	2.75%
Posterior reversible encephalopathy syndrome (PRES)	3	2.75%
Cerebral venous sinus thrombosis	3	2.75%
Shunt malfunction	3	2.75%
Stroke	2	1.83%
Arachnoid cyst with complication	2	1.83%
Idiopathic increased intracranial pressure	2	1.83%
Intraventricular cyst with hydrocephalus	2	1.83%
Hydrocephalus	1	0.92%
Autoimmune encephalitis	1	0.92%
Multiple sclerosis	1	0.92%
Ataxia telangiectasia	1	0.92%
Cerebral involvement of ALL	1	0.92%
Non emergent intracranial lesion		
Headache disorders	26	23.85%
• Migraine	20	18.35%

• Tension-type headache	6	5.50%
Inconclusive diagnosis	14	12.84%
Others; such as dengue fever, epilepsy, chronic sinusitis, and hypertension	11	10.09%

Supporting information Table S3. Breakdown of top three emergent intracranial lesion

Brain tumor (total 13)	
Mature teratoma of the pineal gland	1
Pituitary macroadenoma	2
Ependymoma	1
Medulloblastoma	3
Diffuse astrocytoma of the brain stem	1
Non-germinomatous germ cell tumor of the pineal gland	1
Glioblastoma	2
Germinoma of the pineal gland	1
CNS involvement of the acute lymphocytic leukemia (ALL)	1
Infection (total 13)	
HIV encephalopathy	1
Cryptococcal meningitis	1
Neurocysticercosis	2
Japanese encephalitis (JE)	2
Cerebral toxoplasmosis	1
Pyogenic brain abscess	3
Bacterial meningitis	2
Dengue encephalitis	1
Hemorrhage (total 7)	
Bleeding from arteriovenous malformation	3
Bleeding from cavernoma	1
Bleeding from ruptured aneurysm	1
Unknown cause	2

Supporting information Table S4 Association between red flags and emergent intracranial lesions

Red-flags (n, col %)	Total (N=109)	Emergent intracranial lesion		
		Negative lesions (n=58)	Positive lesion (n=51)	p-value
Acute onset (<3 months)	72 (66.1)	30 (51.7)	42 (82.4)	<0.01
Severe vomiting	65 (59.6)	31 (53.5)	34 (66.7)	0.16
High-risk underlying comorbidities	26 (23.8)	11 (19.0)	15 (29.4)	0.20
Fever	21 (19.3)	8 (13.8)	13 (25.5)	0.12
Focal motor abnormality	20 (18.4)	3 (5.2)	17 (33.3)	<0.01
Changes in mood or personality over days or weeks	20 (18.4)	5 (8.6)	15 (29.4)	<0.01
Altered conscious state	19 (17.4)	4 (6.9)	15 (29.4)	<0.01
Seizures	16 (14.7)	5 (8.6)	11 (21.6)	0.06
Abnormal ocular movements, squint, pathological pupillary responses	16 (14.7)	2 (3.5)	14 (27.5)	<0.01
Increase in severity or characteristics of the headache	14 (12.8)	5 (8.6)	9 (17.7)	0.16
Pain that wakes the child from sleep or occurs on waking	13 (11.9)	8 (13.8)	5 (9.8)	0.52

Ataxia, gait abnormalities, impaired coordination	13 (11.9)	1 (1.7)	12 (23.4)	<0.01
Meningism	12 (11.0)	3 (3.5)	10 (19.6)	<0.01
Occipital headache	11 (10.1)	8 (13.8)	3 (5.9)	0.17
Visual field defects	10 (9.2)	3 (5.2)	7 (13.7)	0.12
Cranial nerve palsies	8 (7.3)	2 (3.5)	6 (11.8)	0.14
Papilledema	7 (6.4)	2 (3.5)	5 (9.8)	0.18
Age < 5 years	6 (5.5)	2 (3.5)	4 (7.8)	0.32
Poor general condition	5 (4.6)	0 (0)	5 (9.8)	0.02
Sudden onset of headache (first or worst ever)	5 (4.6)	1 (1.7)	4 (7.8)	0.18
Worsening of pain with cough or Valsalva maneuver	3 (2.8)	5 (5.2)	0 (0)	0.25
Increased head circumference	1 (0.9)	1 (1.7)	0 (0)	1.00
Change of the character of headache in patients diagnosed with primary headache	0 (0)	0 (0)	0 (0)	--

Supporting information Table S5. Discrimination and diagnostic value for each red flag

Red flags	Sensitivity (%)	Specificity (%)	Correctly Classified	Positive Likelihood Ratio	AuROC (95% CI)
Acute onset (<3 months)	82.4	48.3	64.2	1.59	0.65 (0.56 to 0.74)
Severe vomiting	66.7	46.6	56.0	1.25	0.56 (0.47 to 0.66)
High-risk underlying comorbidities	29.4	81.0	56.9	1.55	0.55 (0.47 to 0.63)
Fever	25.5	86.2	57.8	1.85	0.56 (0.48 to 0.63)
Focal motor abnormality	33.3	94.8	66.1	6.44	0.64 (0.56 to 0.71)
Changes in mood or personality over days or weeks	29.4	91.4	62.4	3.41	0..60 (0.53 to 0.68)
Altered conscious state	29.4	93.1	63.3	4.26	0.61 (0.54 to 0.68)
Seizures	21.6	91.4	58.7	2.50	0.56 (0.49 to 0.63)
Abnormal ocular movements, squint, pathological pupillary responses	27.5	96.6	64.2	7.96	0.62 (0.55 to 0.68)
Increase in severity or characteristics of the headache	17.7	91.4	56.9	2.04	0.54 (0.48 to 0.61)
Pain that wakes the child from sleep or occurs on waking	9.8	86.2	50.5	0.71	0.48 (0.41 to 0.54)
Ataxia, gait abnormalities, impaired coordination	23.5	98.3	63.3	13.6	0.60 (0.54 to 0.67)

Meningism	19.6	96.6	60.5	5.69	0.58 (0.52 to 0.64)
Occipital headache	5.9	86.2	48.6	0.43	0.46 (0.41 to 0.52)
Visual field defects	13.7	94.8	56.9	2.65	0.54 (0.49 to 0.60)
Cranial nerve palsies	11.8	96.6	56.9	3.41	0.54 (0.49 to 0.59)
Papilledema	9.8	96.6	56.0	2.84	0.53 (0.48 to 0.58)
Age < 5 years	7.8	96.6	55.1	2.27	0.52 (0.48 to 0.57)
Poor general condition	9.8	100.0	57.8	-	0.55 (0.51 to 0.59)
Sudden onset of headache (first or worst ever)	7.8	98.3	56.0	4.55	0.53 (0.49 to 0.57)
Worsening of pain with cough or Valsalva maneuver	0	94.8	55.5	0	0.47 (0.44 to 0.50)
Increased head circumference	0	98.3	52.3	0	0.49 (0.47 to 0.51)
Change of the character of headache in patients diagnosed with primary headache	-	-	-	-	-
